# Preliminary findings on psychometric properties of the adolescent story stem profile

**DOI:** 10.3389/fpsyg.2025.1478372

**Published:** 2025-03-11

**Authors:** Yinghao Zhang, Saul Hillman, Mariana Pereira, Katharine Anderson, Richard M. Cross

**Affiliations:** ^1^Anna Freud National Centre for Children and Families, London, United Kingdom; ^2^The University of Hong Kong, Hong Kong SAR, China; ^3^Five Rivers Child Care Ltd., Salisbury, United Kingdom

**Keywords:** adolescent story stem profile, attachment, adolescence, mentalising, affect competence

## Abstract

**Introduction:**

The Adolescent Story Stem Assessment Profile (ASSP) is a newly developed narrative measure aimed at assessing the psychological wellbeing of adolescents. This study investigates the psychometric properties of the ASSP within the British population, with the goal of elucidating its unique strengths and limitations.

**Methods:**

We conducted an exploratory factor analysis on the responses of a community sample of adolescents in the UK (*N* = 182) to identify the underlying factors that reflect adolescents’ internal representations as measured by the ASSP. Following this, we performed a series of analyses on the responses from both the community sample and a high-risk sample of adolescents in care (*N* = 67) to investigate the psychometric properties of the ASSP.

**Results:**

We demonstrated satisfactory internal consistency, construct reliability, and discriminant validity among adolescents with varying levels of risk factors. The findings suggest that the ASSP is a promising tool for measuring mental health in adolescents, providing an economical and practically accessible method for both preventative and clinical applications.

**Discussion:**

Our results yield critical insights into the complex nature of adolescents’ psychological development, highlighting the necessity for tailored measures and interventions that address the diverse psychological needs of this population. Overall, this study represents a significant initial step toward establishing the ASSP as a valuable resource in both research and clinical practice, with implications for future studies aimed at enhancing our understanding of attachment to carers, peer relations, mentalization and affect competences in adolescence. The insights gained from this research underscore the importance of developing assessment tools that are sensitive to the unique psychological experiences of adolescents, ultimately contributing to more effective interventions and support strategies.

## 1 Introduction

Adolescence, defined as the age group of 10–19 years (World Health Organization, 2025), represents a significant transitional phase between childhood and adulthood. During this critical period, individuals experience complex renegotiations of self-concept, family dynamics, social relationships, responsibilities, and aspirations for the future (Moretti and Peled, 2004). The inherent instability of the psychological profile characteristic of this developmental stage increases the likelihood of both internal and external conflicts, which can manifest as emotional and behavioral disturbances. Such disturbances may elevate the risk of developing various mental health issues (Cicchetti and Rogosch, 2002; Casey et al., 2014).

A comprehensive understanding of adolescents’ psychological profiles is essential for promoting effective prevention and intervention strategies in mental health. However, our understanding of adolescents’ internal representations remains somewhat constrained, primarily due to a significant “measurement gap” identified in the assessment of attachment during this developmental stage (Allen, 2008), with most pre-existing measures targeting adults or younger children. In addition, previous research has predominantly focused on disruptive emotions and behaviors, along with their negative consequences for young people (Marques et al., 2011). Widely used measurement scales for assessing adolescents’ mental states, such as the Revised Children’s Anxiety and Depression Scales (RCADS; McKenzie et al., 2019), have primarily been designed for diagnostic purposes. This focus often neglects the potential protective factors and resilience present in adolescents (Bentley and Bucci, 2019). Meanwhile, the social stigma and embarrassment associated with such diagnostic measures can reinforce barriers that prevent young individuals from accessing necessary mental health assessments and services (Radez et al., 2021). Additionally, the clinical nature of the diagnostic measures typically necessitates administration by a qualified clinician, which, in the context of severely limited clinical resources, exacerbates the existing imbalance between the high demand for mental health screening and intervention and the availability of services. This situation underscores the urgent need for the development of more holistic, reliable, and accessible methods for assessing and enhancing wellbeing in adolescents across both clinical and non-clinical settings (Schechter et al., 2007).

Research has increasingly advocated for a holistic approach that considers wellbeing, interpersonal relationships, social competencies, and overall quality of life as critical components influencing mental health outcomes (McCauley et al., 2017). In response to this need, this paper introduces a newly developed narrative measure, the Adolescent Story Stem Assessment Profile (ASSP), aimed at advancing relevant research and clinical practices for prevention and intervention for adolescents’ mental wellbeing. Grounded in Attachment Theory (Bowlby, 1969), the ASSP provides insights into adolescents’ psychological profiles through the lenses of attachment to parents, attachment to peers, affective competencies, and reflective functioning. This approach offers a more comprehensive understanding of the key psychological constructs pertinent to adolescents, thereby facilitating targeted prevention and intervention efforts in mental health.

### 1.1 Attachment in adolescence

Attachment theory, as articulated by Bowlby (1969), elucidates the development of psychological wellbeing through the lens of early interpersonal relationships, offering valuable insights into the psychological constructs of adolescents. According to Bowlby (1969, 1973), infants are innately driven to form close attachments with significant others, typically their primary caregivers, who become the primary attachment figures that play a key role in facilitating the infants’ normal development These primary attachment figures function as a “secure base” and “safe haven,” providing both proximity and support for exploration, as well as fostering adaptive behaviors in children, particularly in the face of perceived threats (Ainsworth et al., 1978).

During the attachment processes, early experience of interactions with primary attachment figure could be incorporated to construct an internal working model (IWM) (Bowlby, 1969 Bowlby, 1973), which encompasses a mental representation of how individuals perceive themselves and their significant others within relational contexts. Evolving from the overall attachment styles, the way in which people cognitively perceive themselves and others, predict and respond to other’s behaviors, and direct future social strategies are all encoded in their IWM. The IWM subsequently informs one’s interaction patterns with others, significantly shaping self-perception and influencing behaviors across various relationships and situations (Pietromonaco and Barrett, 2000). The IWM was hypothesized to undergo fine revisions and refinements with new experience but remain largely stable throughout the lifespan (Bowlby, 1973).

Additionally, increasing evidence highlighted the significant role that the attachment system plays beyond infancy, extending throughout the entire developmental process across the lifespan (Ainsworth, 1989). Research indicates that attachment security remains substantially stable during adolescence (Allen et al., 2004), making it a reliable and meaningful measure of an individual’s psychological profile at this critical developmental stage.

During adolescence, individuals with better attachment qualities are generally better supported in establishing autonomy and exploring their environments (Therriault et al., 2021). This support is crucial for satisfying the key developmental needs that emerge during this period (Allen et al., 1994). Numerous studies have demonstrated that higher levels of attachment security during adolescence are associated with enhanced psychological wellbeing across various dimensions (Oldfield et al., 2016). These dimensions include cognitive and socioemotional competence (Allen et al., 2003), improved stress-coping mechanisms (Howard and Medway, 2004), greater self-satisfaction (Armsden and Greenberg, 1987), reduced levels of mental health issues (Flykt et al., 2021), and higher quality relationships with parents, peers, and romantic partners (Furman et al., 2002). Conversely, lower levels of attachment security have been linked to an overall increase in emotional and behavioural difficulties during adolescence (Wambua et al., 2018), including externalizing and internalizing disorders (Fearon et al., 2010; Groh et al., 2012), eating disorders (Jewell et al., 2016) and suicidality (Fergusson et al., 2000). This underscores the importance of fostering good attachment systems throughout development to promote psychological outcomes.

In the following section of the paper, we will explore four key psychological constructs related to attachment: attachment to parents, attachment to peers, mentalization, and affect competence. We will emphasize the significant roles these constructs play in fostering psychological wellbeing, drawing upon the principles of attachment theory.

#### 1.1.1 Attachment to parents

Parent–child attachment, which develops from early infancy, remains a crucial factor for wellbeing during adolescence (Armsden and Greenberg, 1987). The attachment patterns between children and their caregivers exhibit substantial continuity from early childhood into adolescence, despite a reduction in the amount of time spent with parents during this developmental stage (McCormick and Kennedy, 1994; Allen et al., 2004). While being a largely unconscious process, the security or insecurity in children’s attachment relationships bears significant implications of their other interpersonal relationships across development (Schneider et al., 2001).

As adolescents navigate the increased tension between their fundamental needs for autonomy and the attachment bond that serves as a secure base, the dynamics of child-caregiver relationships can become more complex. However, this does not imply that an adolescent’s desire to establish an independent sense of self is inherently contradictory to the strong attachment they maintain with their caregivers (Allen and Land, 1999; McElhaney et al., 2009). In fact, secure child-caregiver attachment provides a stable foundation that enables adolescents to focus on exploring and mastering their environments (Sroufe, 2005). This secure attachment remains essential for fostering better psychological outcomes during this critical period of development (McElhaney et al., 2009).

Empirical evidence indicates that poor parent–child attachment quality during adolescence significantly increases the risk of developing mental health problems, as demonstrated in a two-year longitudinal study (Bannink et al., 2013). Conversely, secure attachment relationships between parents and children are associated with a range of positive outcomes, including fewer symptoms of depression following stressful life events (Aliri et al., 2019), lower levels of generalized anxiety disorder symptoms (van Eijck et al., 2012), and enhanced cognitive, emotional, and social competence (Moretti and Peled, 2004). Importantly, strong attachment relationships between children and their caregivers also promote positive interactions and relationships with peers and friends. This supportive network is crucial for adolescents’ mental wellbeing, as it enables them to seek assistance and feel secure in various contexts (Delgado et al., 2022). Thus, fostering secure attachment during adolescence not only benefits the individual but also enhances their social relationships, contributing to overall psychological health.

#### 1.1.2 Attachment to peers

Given that adolescents increasingly spend time with peers in social environments such as schools, peers play a significant role in their development and adjustment (Rubin et al., 2006). During this developmental stage, the primary source of support may shift from parents to peers and romantic partners as adolescents seek greater independence (Fraley and Davis, 1997). In this context, peers can serve as alternative or supplementary attachment figures, complementing the role of parents in relevant situations (Meeus et al., 2002; Hazan and Shaver, 1987; Goossens et al., 1998). The mental representations of relationships, which are based on internal working models, initially develop from interactions with caregivers during early life. Consequently, the relationship between parent–child and peer-child attachment has been proposed, although findings regarding the nature of this linkage have been mixed (Meeus et al., 2002; Lamborn et al., 1991; Alcaide et al., 2023). Nevertheless, research indicates that adolescents who exhibit high attachment security in both parent and peer relationships tend to demonstrate the best adjustment outcomes (Laible et al., 2000).

Furthermore, several studies have highlighted the importance of peer attachment alone in relation to adolescents’ psychological health outcomes (e.g., Armsden and Greenberg, 1987; Cotterell, 1992). For instance, the quality of peer attachment has been shown to mediate the negative effects of stressful life events on adolescents’ psychological wellbeing, particularly among girls (McMahon et al., 2020). The quality of peer attachment also appeared to be strongly correlated with the positivity of self-esteem and self-concept (Wilkinson, 2004). A meta-analysis synthesizing data from 24 studies reported a positive correlation between higher quality peer attachment and increased self-esteem (Gorrese and Ruggieri, 2013). Additionally, secure attachment to peers has been associated with a lower risk of developing depressive symptoms and more positive cognitive coping styles (Chen et al., 2019). Overall, attachment to peer plays a crucial role in shaping psychological outcomes and interacts in complex ways with parental attachment, ultimately influencing the overall developmental trajectory of adolescents.

#### 1.1.3 Mentalization

Mentalization, or reflective functioning, is a more recent and integrative concept that grew out of the attachment theory. It offers a complementary perspective to traditional attachment framework and can be jointly considered with attachment to provide a holistic insight into adolescents’ psychological constructs (Fonagy et al., 2015). Mentalization refers to the ability to understand the intentional, motivational and emotional mental states in self and others, thereby making sense of interpersonal feelings, thoughts and behaviors (Fonagy et al., 2002). The initial formation of these internal representations is largely dependent on the early child-caregiver relationship (Fonagy and Allison, 2012), and becomes gradually strengthened and stabilized throughout development while the individual finds themselves an appropriate place in relation to others in the social world (Gergely, 2001).

An adequate mentalizing capacity is suggested to be important for developing appropriate self-control, affect regulation, social functioning, cognitive abilities, and consistent agency (Fonagy and Target, 1998). Abundant evidence from theoretical and empirical research has been demonstrating the link between compromised mentalization and various psychological difficulties in adolescents, including self-harm (Rossouw and Fonagy, 2012), affective disorders (Murri et al., 2017) and suicidality (Pompili et al., 2017), highlighting the essential role of mentalization in psychological wellbeing. Recent studies also proposed the health-promoting role of mentalization in non-clinical populations (Luyten et al., 2020), including facilitating the development of an integrated sense of self (Taubner, 2015), allowing for better processing of affective arousal under stress (Ballespi et al., 2019) and enhancement of emotion regulation (Schwarzer et al., 2021).

#### 1.1.4 Affective competency

Emotional or affective competency is a crucial psychological construct that enables individuals to accurately perceive their own emotions and those of others, thereby utilizing these emotions to facilitate thinking (Salovey and Sluyter, 1997). Higher affective competency can positively predict adolescents’ adjustment to environmental changes and overall mental wellbeing (Petrides et al., 2016; Gómez-Baya et al., 2017; Fernández-Berrocal and Extremera, 2016).

Given its central role in socioemotional functioning, affective competency is essential for maintaining interpersonal relationships, extending beyond mere attachment (Jiménez-Rodríguez et al., 2022). Insecure attachment can lead to maladaptive responses to threats and stress, both within and outside primary attachment relationships, resulting in poorly adjusted emotional responses (Mikulincer and Shaver, 2019). During adolescence, secure attachment is associated with better self-concept formation, lower levels of psychopathological symptoms, and reduced engagement in risk behaviors. Importantly, these associations are moderated or mediated by affective regulation capacities and the presence of affective difficulties (^34,87^).

It has been suggested that adolescents with varying attachment styles may employ different affect regulation strategies in response to emotional arousal. For instance, individuals with anxious attachment may experience heightened worry in the absence of primary attachment figures, while those with avoidant attachment may adopt emotional distance as a coping mechanism, rather than relying on significant others (Brenning and Braet, 2013). Given the intricate interplay between attachment and affective competency, a multimethod approach is recommended to measure these constructs separately and to explore their underlying mechanistic links (Brumariu, 2015).

#### 1.1.5 Attachment in young people in care

An important population at a higher risk for attachment deficits and, consequently, psychological difficulties is adolescents in care (Miranda et al., 2019). Placement in either residential or foster care—often referred to as institutionalization—serves as a supportive intervention aimed at safeguarding the wellbeing of children and adolescents who have experienced early parental loss, abandonment, or removal from their biological families due to adverse circumstances such as neglect, abuse, or parental incapacity. Ideally, this intervention is intended to be temporary, with the goal of either reuniting the child with their original caregiver or facilitating adoption if the biological caregivers remain unable to provide adequate care.

However, the necessity for young people to be placed in care often indicates prior experiences of abusive or neglectful maltreatment, which can result in psychological trauma that may persist throughout their lives (Bellamy, 2008; Doyle, 2013). Due to the high prevalence of childhood trauma and the severely dysfunctional early caregiving environments, young people in care are considered to be at an elevated risk for psychological difficulties (Greeson et al., 2011; Joseph et al., 2014). Research indicates that adolescents in foster care experience more emotional and behavioural challenges compared to their peers in community samples (Pears et al., 2010). This disparity is linked to increased risks for psychological difficulties encompassing many aspects that can persist throughout their lifespan (Kim and Cicchetti, 2010).

The suboptimal psychological wellbeing observed in young people with experiences in care can be attributed, at least in part, to the significantly higher rates of insecure attachment styles (Bifulco et al., 2017). The frequent turnover of caregivers during the care experience—particularly in the early years—can lead to significant disruptions in the formation of attachment bonds between the child and their designated primary caregivers. This instability not only undermines the development of new attachment relationships but can also damage any pre-existing attachment bonds, resulting in delayed or maladaptive development of the child’s internal working model (Van IJzendoorn et al., 2011). Such disruptions can hinder the optimal development of emotional and behavioural regulation skills (Cicchetti and Toth, 2015) and may lead to difficulties in forming new attachment bonds in the future (Joseph et al., 2014), negatively affecting the attachment bonds with parents and peers. Insecure attachment is associated with decreased psychological functioning, including critical skills, reflective abilities, social and academic competence, adaptive responses to stress, and the capacity to trust adults (Muzi and Pace, 2021). These findings underscore the necessity of effectively assessing and promoting attachment-related processes among young people in care (Zegers et al., 2008).

### 1.2 Existing measures for adolescents’ internal representations

Stemming from and closely related to the attachment theory, one’s attachment to parents and peers, mentalization capacity and affective competency may interact in different ways (Goodall et al., 2012), leading to different psychological adjustment outcomes. Measuring these psychological constructs holistically can provide a distinct and detailed overview of an adolescents’ mental profile, revealing both potential vulnerabilities and resiliencies in the individual. Consequently, measuring attachment and other relevant constructs while understanding the interplay between them could offer critical insights into the complete picture of the mental states in adolescents (Tanzilli et al., 2021).

To date, some interview approaches have been developed based on attachment theory to access the internal representation in adolescents [e.g., Children Attachment Interview, CAI; Shmueli-Goetz et al., 2008; the Attachment Style Interview for Adolescents (ASI-AD); Bifulco et al., 2017]. In some cases, measures that are best suitable for adults (e.g., Adult Attachment Interview, AAI; George et al., 1985) or younger children (Friends and Family Interview, FFI; Steele and Steele, 2005) can also be used to measure attachment in adolescents. Additionally, a range of self-report questionnaires (e.g., Inventory of Parent and Peer Attachment-Revised, IPPA-R; Gullone and Robinson, 2005) were designed to measure attachment in adolescents. However, there is only very limited evidence on the structural and content validity of these measures, with no existing measure shown to have satisfactory psychometric properties (Jewell et al., 2016). Besides, there is no or only a weak correlation between the results synthesized by different measures, indicating that at least some domains of attachment were not adequately captured in these measures (Allen, 2008).

On the other hand, mentalization is a relatively new concept that has only became increasingly popular in the last decade. Well established measures for mentalization capacity in children and young people has only been developed comparably recently (Sharp et al., 2022), with some of them relying heavily on the transcripts of the attachment interview, providing little additional materials on mentalization per se (Chow et al., 2014). Consequently, existing measures for mentalization faces same problems as attachment measures, such that there still lacks systematic, valid measures that can capture mentalization capacity in adolescents satisfactorily (Chow et al., 2017).

Affective competency is a broadly defined term that encompasses various dimensions, ranging from fundamental skills such as emotion labeling and recognition to more complex affect regulation strategies, including the ability to understand and infer emotional states in oneself and others [or broadly emotion intelligence (Bar-On and Parker, 2000; Stough et al., 2009; Keefer, 2015)]. Despite the increasing interest in this construct, significant inconsistencies in measurement outcomes persist, largely due to the absence of a clear definition that delineates which specific skills should be included as part of affective competency at different developmental stages (Roberts et al., 2010). In addition, self-report measures of affective competencies may be particularly susceptible to ambiguities, as they can be influenced by varying cognitive and behavioral patterns, as well as individual differences in intelligence (De Los Reyes, 2011). These factors can lead to response biases and both conscious and unconscious self-misinterpretations, which may obscure the true effects being measured (Dunning et al., 2004). Indeed, research has shown that adolescents often struggle to accurately assess their own emotional competencies (MacCann et al., 2010).

The heterogeneity as well as the sub-optimal quality of results generated by different measures could be caused by several reasons. Firstly, attachment and its relevant factors are concepts that belong to higher-order cognitive domains, such that it is difficult to assess these abstract concepts conclusively and explicitly (Jewell et al., 2016). Breaking them down into lower order subconstructs such as emotion regulation, social competencies and reward processing and assessing them in a broader social context could be more helpful, since the subconstructs are comparably easier to test.

Meanwhile, developmental neuropsychology research suggested that language and cognitive ability could undergo dramatic changes within a short period of time during adolescence (Burgaleta et al., 2014). The inconsistent cognitive and psychological constructs of adolescents might further complicate their responses to relevant measures as different individuals might have different capabilities to respond to the same measure. This might bring further concerns regarding how data generated from these measures should be interpreted, and novel measurements should take the latent construct of adolescence into consideration.

To conclude, although the attachment theory provides a good theoretical framework for assessing adolescents’ psychological wellbeing, no gold standard measures of attachment and mentalization exist given the volatile psychological profile of for this developmental phase (Bosmans and Kerns, 2015). This highlights the lack of novel measures with adequate psychometric properties. The Adolescent Story Stem Profile (ASSP), a new measurement tool developed basing on the attachment theory, could fill this void.

### 1.3 The adolescent story stem profile (ASSP)

The ASSP is an online narrative measure that assesses psychological constructs of adolescents by asking them to complete a story following a given prompt (Hillman et al., 2020). As a narrative measure, it presents the adolescents with the beginning of a story that consists of an everyday scenario with a certain degree of conflict using animated videos. By inquiring about the young person’s thoughts about the emotionally triggering situation and asking them to complete the story, the ASSP explores how adolescents regulate their affect, make sense of themselves and others in different social situations, and resolve conflicts.

Based on the Story Stem Assessment Profile (SSAP; Hillman et al., 2020), a well-established narrative measurement designed for younger children, the ASSP is adapted to be age-appropriate for adolescents in order to provide a reliable measure for this developmental stage and fill the gap in research. Similar to the Story Stem Assessment Profile (SSAP), participants are not posed direct questions regarding their family dynamics. Instead, the responses generated from the ASSP are based on animated daily situations in a narrative manner, serving as a valuable gateway into understanding the internal working models of young individuals. Specifically, the six animated videos in the ASSP consist of both high-arousing and low-arousing story stem challenges in a range of daily social situations (e.g., having arguments with friends, witnessing parents’ conflicts at home). The multiple-choice questions associated with each story explore the young people’s attachment to family and peers, emotion regulation, self-esteem and mentalization when they confront different challenges. As compared to the unidimensional diagnostic measures, the ASSP could tap into multiple dimensions in the young people’s internal representation, providing a more precise and complete assessment of the problem-solving strategies and socioemotional functioning of young people (Robinson, 2007).

With reference to the attachment theory, it has been proposed that story-based narratives of young people could sufficiently reflect their own social and behavioral experience (Page, 2001). In particular, their internal working models and psychological representations can be reflected by their thoughts and feelings toward the characters in the stories (e.g., how the young people think the story characters would respond to conflicts might reflect the young people’s own strategies toward conflicts). Consequently, the narrative measure could provide a gateway into young people’s emotion regulation capacity (Appelman and Wolf, 2003), representation of the self (Toth et al., 2000), attachment style (Green, 2003) and family functioning (Poehlmann and Huennekens, 2003). On the other hand, risks of various psychological difficulty could be revealed by story stem measures as they engage young people in constructing and reviewing the resolution of the stories (Robinson, 2007). For example, rumination and low mood in the young people could be indicated by their over-generalized and script-like negative responses to stories with different themes. Narrative measurements have been theoretically and empirically validated in both typically developing and maladaptive populations with various demographic backgrounds.

The ASSP has multiple advantages compared to other measurements. Given that it can be accessed and completed online by the young people independently, there is more flexibility in the time and location that the assessment is carried out. Without the need of interacting with a professional, the ASSP requires less resources and is less time-consuming in comparison to interviews and observational methods. Consequently, the ASSP is easy to administer and score, and has better accessibility (Lundahl et al., 2014). The ASSP could be particularly useful in maltreated and disadvantaged populations as it would often be difficult to obtain accurate and unbiased information from the schoolteachers and caretakers of these young people (Plokar and Bisaillon, 2016). In sum, the ASSP could provide a better understanding of the young people’s internal representations in general by directly seeking information from them (Lundahl et al., 2014).

Additionally, the ASSP takes the limited verbal and cognitive capacities of developing young people into consideration, especially those with mental health difficulties. It demonstrates the story stems with animated videos that are easy to understand. By doing so, the ASSP probes the thoughts and feelings of young people in a more comprehensive way than self-report measures. Additionally, young people might consciously or unconsciously withhold some of trauma and adverse experiences as they might normalize such experiences or fear to report them (Macfie et al., 2001). These subtle constructs could be potentially revealed by the young people’s responses to the story stem in the ASSP. As the story stem measure only taps into the young people’s internal world through imaginative characters, it is less threatening and intrusive in nature, so that it reduces the risks of disengagement of traumatized individuals (Nadar, 2011). Given that, the ASSP could be useful in a wide range of populations in school, foster and clinical settings.

### 1.4 Aim and hypotheses

The primary aim of this study is to rigorously validate and assess the psychometric properties of the Adolescent Story Stem Profile (ASSP) by analysing data collected from two distinct samples of British adolescents aged 10–17: a low-risk sample recruited from UK secondary schools and a high-risk sample comprising adolescents with prior experience in foster care. Through a comprehensive series of statistical analyses, we will evaluate the factor structure, internal consistency, convergent validity, and discriminant validity of the ASSP. Our goal is to establish the robustness and reliability of the ASSP as a valid measure of adolescents’ inner representations, with the potential for practical application in both clinical and non-clinical settings. We hypothesize that the Attachment Security Scale for Parents (ASSP) will exhibit satisfactory psychometric properties, including a well-defined factor structure, strong internal consistency, and evidence of convergent validity. Additionally, we anticipate observing differential responses between low-risk and high-risk samples when assessed with the ASSP, which would provide support for its discriminant validity. However, given that the ASSP is a newly developed tool, our hypotheses are primarily exploratory in nature.

## 2 Methods

### 2.1 Sample

In total, data was collected from 326 young people aged from 10 to 17 years. After data collection, 77 young people were excluded from the final analysis due to incomplete data entries. Consequently, the final sample comprised of 249 young people (M_age_ = 14.1, SD = 1.56, *N*_female_ = 143).

The community low-risk sample involves 182 adolescents (*N*_female_ = 111, Mean age = 13.86, SD = 1.48) from two secondary schools in different cities in the UK. All participants were living with their biological family and had not had previous experiences in care. Both secondary schools are large and both and ethnically diverse, however, further demographic information was not available on this sample. Inclusion criteria were English fluency and the ability to complete the online task independently. Teachers at the secondary schools were consulted to make sure the participating adolescents met the inclusion criteria. The data collection was conducted in 2023.

The high-risk sample consisted of 67 young individuals (33 males and 34 females) aged 10–17 years (M = 14.8, SD = 1.8). Participants in this group were either in foster care or residential care, with a mean age of entry into care of 7.9 years (SD = 3.3). At the time of data collection, participants were enrolled in either mainstream secondary schools (59.3%) or Special Educational Needs and Disabilities (SEND) educational support structures (40.7%) in the UK. Among the high-risk sample, over one-third (35.7%) were receiving active psychological or psychiatric therapy, 21.4% were on medication, and 2.9% had either currently been admitted to or had previously been admitted to a psychiatric unit. In general, the high-risk sample has additional adverse experience around their development environment and was considered to be more vulnerable to psychopathology compared to the low-risk, community sample. The inclusion criteria for the high-risk sample were consistent with those of the low-risk sample. A clinician who worked with the participants ensured that all individuals met the specified inclusion criteria. Data from the high-risk sample were collected between 2019 and 2021.

### 2.2 Measures

#### 2.2.1 The adolescent story stem profile (ASSP)

The ASSP is a narrative measurement for assessing internal representations within adolescents. It was adapted from the SSAP (Hillman et al., 2020) which is a similar narrative measurement developed earlier and validated in younger children.

The ASSP is composed of six animated videos. Participants were asked to watch the videos, in which the main character was gender-matched to the participants identified gender. Each video tells the beginning of a story containing a common social situation with a certain level of conflict and is therefore likely to elicit emotional responses in the participants. After watching each video, participants were given a series of multiple-choice questions that ask them about how they think the characters in the video would feel and react. Participants’ thoughts and feelings toward the story characters are proposed to mirror their own ways of thinking when encountering similar situation in their daily lives (Page, 2001).

In total, the ASSP contains 90 multiple-choice questions that can be categorized into four subscales: (A) Mentalization, (B) Attachment, (C) Affective competency, and (D) Story-self relevance. Under each subscale, questions can be further divided into specific themes that examine several functional aspects. Further details on ASSP sub-divisions are in Table 1. See Appendix A for summaries of the six ASSP stories and themes examined in each story (Table 2).

**Table 1:** Summary of question types in four ASSP subscales.

A. Mentalization (36)	B. Attachment (30)	C. Affective competency (12)	D. Story-Self correlation (12)
a1 Accuracy of emotion regulation (30) a1.1 in self (12) a1.2 in parents (10) a1.3 in peers (8)	b1 Relationship with peers (12) b1.1 peer awareness (6) b1.2 peer communication (6)	c1 Affect regulation speed (6)	d1 Story-Self relevance (6)
a* global mentalizing-others (categorical code)	b2 Relationship with parents (12) b2.1 parent awareness (6) b2.2 parent communication (6)	c2 Affect arousal strength (6)	d2 Strength of own feelings triggered by story (6)
	b* global parent-attachment (categorical code)		

The four columns comprise the four subscales of the ASSP, with divisions of further specific themes.

**Table 2:** Summary of the scenarios depicted in ASSP and example questions.

Story	Summary of scenario	Example question
1	Alex suddenly walked out of the living room, where they had been sitting with their parents went up to their bedroom, and slammed the door shut.	a1.1: What do you think Alex might feel when they…? a1.2/a1.3: What do you think Alex’s parents/friends might feel when…? a*: How do you think Alex make sense of their parents? b1/b2: After…, do you think Alex talk to their parents/friends? b1/b2: Do you think Alex’s parents/friends know what is bothering them? b*: What do you think Alex’s parents might have done? c1: How do you think Alex might feel much later on that day? c2: How strongly does Alex feel their emotions during the event? d1: How much did watching this video make you aware of your own thoughts and feelings? d2: How strongly did you feel when you were reminded of your own experience?
2	Alex is given a certificate at school and goes home with it.	
3	Alex gets suspended from school and then comes home.	
4	Alex approaches their friends, asking them whether they would like to do something together. While one agrees, the other one says they are busy doing something else.	
5	Alex’s friends came around and they went out with them. At the end, something happened to Alex, and Alex was sitting on a bench as if either ill or hurt or upset.	
6	Alex heard their parents having an argument when they entered the room.	

The indices of themes are same as those in Table 1.

#### 2.2.2 The reflective function questionnaire for youths (RFQY-5)

The Reflective Function Questionnaire for Youth (RFQY-5) is a 5-item self-report measure assessing mentalizing function in adolescents, which was shortened from the original 46-item reflective function questionnaire for youths using item response theory analyses (RFQY, Sharp et al., 2022). The RFQY-5 asks participants to rate their responses on a 6-point Likert scale ranging from “strongly disagree” to “strongly agree,” and a higher total score indicates better reflective function (e.g., I believe that people can see a situation very differently based on their own beliefs and experiences). The construct validity of the RFQY-5 was demonstrated to be satisfactory in 186 healthy adolescents and 100 inpatient adolescents and was therefore considered to be an effective, easy-to-administer measure for reflective function in both healthy and at-risk adolescents (Sharp et al., 2022). The RFQY-5 was used to examine the content and construct validity of the ASSP from the mentalization perspective, as mentalization is closely linked to the theoretical rationale of the ASSP.

### 2.3 Procedure

This study was approved by the UCL Research Ethics Committee (Approval ID number: 19513/003).

For the low-risk sample, participants were organized to complete the tasks in cluster rooms during school time. Each participant was allocated an account and a password to access the task online. After logging in, participants were first asked to sign an online consent and fill in their age and gender of identification, so that a gender-matched version of the ASSP could be allocated to them. After completing the ASSP, they were asked to complete the RFQY-5. The participants completed all tasks independently, with the facilitation orchestrated by schoolteachers in a consistent order. Notably, the participants always completed the ASSP first, as the primary aim of the study was to examine the internal psychometric properties of the ASSP. This prioritization was particularly important given the potential for halfway withdrawal or noncompliance among the young participants, ensuring that the ASSP data was collected reliably. Furthermore, we do not perceive any disadvantages arising from the lack of counterbalancing in this context. The RFQY-5 task is notably brief and focuses on only one out of many aspects measured by the ASSP. Given the short and non-stimulating nature of the RFQY-5, the risk of order effects is considered to be minimal. Remote support was provided by the researchers. The assessment process took around 40 min to complete.

For the high-risk sample, the ASSP tasks were completed as a part of a wider collaborative study carried out by the Anna Freud Centre and Five Rivers Child Care (FRCC). First, a battery of questionnaires which included the Strength and Difficulty Questionnaire (SDQ: Goodman, 1997) and other measures of adversity, dissociation, and attachment were administered in a wider sample of adolescents-in-care to examine their psychological profile. Professionals in the FRCC Assessment and Therapy team identified a subsample of adolescents with high-risk for psychological difficulty who scored above the clinical cut-off on the SDQ. A letter of informed consent was sent to the identified adolescents’ Local Authority and supervising social workers, who were given 2 weeks to opt-out from the allocated assessments. After the informed consent was obtained from the delegated authorities, a further detailed information sheet about the study were sent to the foster carers of the participating adolescents. The assessment was only carried out for adolescents whose responsible authority had given consent to participate in the study, and whose foster carers agreed that the adolescent would be able to participate. Relevant resources and support were provided accordingly and the ASSP tasks were carried out independently.

#### 2.3.1 Statistical analysis

All analyses were performed with R (version 4.2.0) and R Studio (version 2022.12.0 + 353) software.

The psychometric properties of the ASSP were tested in the first part of the analysis. An exploratory factor analysis was performed using the maximum likelihood method of extraction and the varimax method of oblique rotation to examine the factor structure of the ASSP. According to MacCallum et al. (1999), the sample size of 249 is suggested to be sufficient for factor analyses if the participant to variable ratio is >3:1 (78 out of the 90 questions in the ASSP were included for the EFA, such that a minimum of 234 participants were required). To examine the reliability of the ASSP, the internal consistency was tested using Cronbach’s *alpha* (Cronbach, 1951). To test the convergent validity of the ASSP, cross-measure correlations (Pearson’s *r*) between ASSP scores and the RFQY-5 score were computed for participants in the low-risk group.

The second part of the statistical analyses aimed to establish the discriminant validity of the ASSP while exploring how high-risk and low-risk adolescents responded differently to ASSP questions. A further power analysis was conducted using G* Power (Faul et al., 2007) to calculate the sample size required for the group comparisons. Assuming a similarly large effect size (*d* > 0.8), alpha level = 5% and desired power = 80% to previous studies, the minimum sample size was calculated to be 84 (42 individuals per group) for comparisons between the high-and low-risk samples (Cohen, 1992). Normality was assumed for both low-and high-risk samples given the sample sizes were both larger than 40 (Ghasemi and Zahediasl, 2012). Group differences between the high-risk and low-risk samples were tested individually for the 13 scores resulted from the 13 functional aspects in the ASSP using independent t-tests. Additionally, group differences were also t-tested for the four subscale scores.

## 3 Results

### 3.1 Exploratory factor analysis

The Kaiser-Meyer-Olkin measure showed that the initial model reached criteria for sampling adequacy (overall MSA = 0.74) and the Bartlett’s test of sphericity demonstrated sufficient correlations between ASSP items for EFA (*p* < 0.01). The initial factor extraction using parallel analysis revealed five factors with eigenvalues >1. However, a decision was made to retain three factors for further analysis after considering the result of the scree plot analysis and the interpretability of the factors (Tabachnick and Fidell, 2014). These three factors accounted for a cumulative variance of 52%. Considering the space limitation, a descriptive summary of the key findings is presented here, and the complete EFA factor loading table can be found in the Appendix B.

Factor 1 labeled “Story-self Relevance” consisted of items related to the perceived relevance of the scenario depicted in the story to the participant’s own experience and accounted for 10% of total variance. All 12 items focusing on the story-self relevance loaded on this factor with relatively high loadings, indicating a good correspondence to the pre-defined subscale D.

Factor 2, named “Attachment,” represented items measuring parent and peer attachment. Most of the items (20 out of 24) under the pre-defined Attachment subscale loaded on this factor, together accounting for 18% of the total variance.

Factor 3 is labeled as “Mentalization” and comprised items measuring mentalization related functions. It accounted for 24% of total variance. Items in both Subscale A Mentalization (10 out of 18) and Subscale C Affective Competency (5 out of 12) loaded on this factor. In comparison to the previous two factors, a smaller proportion of items in Mentalization and Affective Competency subscales contributed to this factor. This was particularly notable in themes a1 (emotion recognition accuracy in self), and c2 (affect arousal under conflicts), where only 1/2 and 1/3 items had considerable loadings, respectively. Notably, there was a negative correlation between the items under the theme c1 (affect regulation effectiveness) and other items.

### 3.2 Internal consistency

As the three retained factors in the exploratory factor analysis aligned well with the pre-defined subscale structure of the ASSP, it was decided that the four subscales provided a reasonably clear division for different psychological aspects measured by the ASSP. Given that, the internal consistency was calculated for each subscale using Cronbach’s alpha and the results are shown in Table 3. The internal consistency of the ASSP was generally satisfactory (mean *alpha* = 0.76), indicating good reliability (De Vellis, 2003).

**Table 3:** Reliability coefficients of the ASSP as shown by Cronbach’s alpha.

Subscale	Cronbach’s *a*	95% CI	
		*LL*	*UL*
A. Mentalization	0.74	0.70	0.79
B. Attachment	0.75	0.70	0.79
C. Affective competency	0.63	0.55	0.69
D. Story-self relevance	0.90	0.88	0.91

CI, confidence interval; LL, lower limit; UL, upper limit.

### 3.3 Cross-scale correlation

The Pearson cross-scale correlation for between four ASSP subscales is shown in Table 4. This was computed using the complete sample (*N* = 249). To further establish the validity of the ASSP, the correlation was also computed for ASSP subscales and RFQY-5 using data from the community sample (*N* = 182). All correlations between ASSP subscales were significant at *p* < 0.01, but the scale of the correlations were relatively small (average *r* < 0.3). None of the ASSP subscales was found to be significantly correlated with the RFQY-5. Similarly, the magnitudes of the correlation between ASSP subscales and RFQY-5 were also not robust.

**Table 4:** Pearson’s correlation coefficient between ASSP subscales and RFQY-5.

Variable	*n*	*M*	*SD*	1	2	3	4	5
1. A Mentalization	249	2.02	0.40	—				
2. B Attachment	249	2.04	0.39	−0.26^**^	—			
3. C Affective competency	249	2.86	0.37	−0.12^*^	0.32^**^	—		
4. D Self-story relevance	249	2.03	0.63	−0.24^**^	0.20^**^	18^**^	—	
5. RFQY-5	182	4.53	0.72	−0.03	−0.12	0.10	0.00	—

^*^p < 0.05, ^**^p < 0.01.

### 3.4 Group comparison: testing the validity of ASSP and further explorations

There was no significant difference between the two risk groups’ total scores on the A (Mentalization) subscale. Under the Mentalization subscale, significant differences was found between the high-risk and low-risk group’s performances on emotion recognition accuracy in parents (theme a1.2) *t*(247) = −1.13, *p* < 0.01, and peers (theme a1.3) *t*(247) = −3.32, *p* = 0.02, where the high-risk group (M_a1.2_ = 1.87, SD_a1.2_ = 0.20; M_a1.3_ = 1.89, SD_a1.3_ = 0.20) outperformed the low-risk group (M_a1.2_ = 1.75, SD_a1.2_ = 0.35; M_a1.3_ = 1.82, SD_a1.3_ = 0.27). On contrary, the low-risk group (M = 2.74, SD = 1.37) had a significantly better performance on positive mentalizing frequency (theme a*) than the high-risk group (M = 2.28, SD = 1.48), *t*(247) = 2.19, *p* = 0.03.

For the B (Attachment) subscale, a significant difference was found in the total scores between the low-risk (M = 2.07, SD = 0.42) and high-risk (M = 1.96, SD = 0.30) groups, *t*(247) = 2.40, *p* = 0.02, that the low-risk group had better attachment responses in general. However, no significant difference was found in any theme under the Attachment subscale. The difference in the two group’s frequency of selecting secure attachment behavioral patterns (theme b*) was borderline-insignificant, *t*(247) = 1.77, *p* = 0.08.

The high-risk group (M = 3.04, SD = 0.35) had a significant higher total score in subscale C (Affective Competency) than the low-risk group (M = 2.80, SD = 0.35), *t*(247) = −5.00, *p* < 0.01. This was relevant to the significant higher score of the high-risk group (M = 2.92, SD = 0.53) than the low-risk group (M = 2.53, SD = 0.44) in their affect regulation speed (theme c1), *t*(247) = −4.75, *p* < 0.01.

Although the high-risk group scored higher in subscale D (Story-self Association) and the relevant themes in general, no significant difference was found.

No significant difference was found in other themes of the ASSP. The effect sizes for all the between-group comparisons were small to moderate (Cohen’s *d* ranging from 0.07–0.57) except for Affective Competency (*d* = −0.71). The full results are shown in Table 5.

**Table 5:** Independent *t*-test comparing the differences in the ASSP performances of high- and low-risk groups.

Logistic parameter	Low-risk *n* = 182		High-risk *n* = 67		*t*	*p*	Cohen’s *d*
	*M*	*SD*	*M*	*SD*			
A. Mentalization	2.04	0.41	1.98	0.36	1.08	0.28	0.32
a1.1. Emotion recognition in self	1.83	0.21	1.86	0.17	−1.13	0.26	0.14
a1.2. Emotion recognition in parent	1.75	0.35	1.87	0.20	−3.32	0.01^*^	−0.15
a1.3. Emotion recognition in peers	1.82	0.27	1.89	0.20	−2.39	0.02^*^	−0.37
a* Positive mentalization frequency	2.74	1.37	2.28	1.48	2.19	0.03^*^	−0.30
B. Attachment	2.07	0.42	1.96	0.30	2.40	0.02^*^	0.30
b1.1. Peer awareness	2.41	0.48	2.39	0.53	0.38	0.70	0.57
b1.2. Peer communication	2.24	0.54	2.20	0.56	0.56	0.58	0.08
b2.1. Parent awareness	2.13	0.49	2.02	0.53	1.54	0.12	0.23
b2.2. Parent communication	2.00	0.50	1.91	0.51	1.29	0.20	0.19
b* Secure attachment frequency	1.58	1.17	1.19	1.26	1.77	0.08	0.25
C. Affective Competency	2.80	0.35	3.04	0.35	−5.00	0.01^*^	−0.71
c1. Affect regulation speed	2.53	0.44	2.92	0.53	−4.75	0.01^*^	−0.62
c2. Affect arousal under conflicts	3.06	0.49	3.17	0.58	−1.53	0.13	−0.24
D. Story-Self Association	2.01	0.61	2.08	0.69	−0.70	0.48	−0.11
d1. Story-self relevance	2.55	0.89	2.65	1.02	−0.74	0.46	−0.11
d2. Story impact on own emotion	1.47	0.43	1.50	0.43	−0.52	0.60	−0.07

^*^measured as conceptual categories.

## 4 Discussion

This study aimed to examine the psychometric properties of the ASSP in clinical and community samples of young people aged 10–17 years in the UK, while exploring the differences in the psychological constructs in clinical and non-clinical adolescents with their responses to the ASSP. Mixed results were obtained for the psychometric properties of the ASSP. Additionally, several important findings regarding how young people at different risks for psychological difficulties perceive both themselves and their relationships were uncovered. These findings partially supported the broader usage of the ASSP in the future.

### 4.1 Psychometric properties of the ASSP

According to the EFA results, all ASSP items generating ordinal responses met the criteria for sampling adequacy, meanwhile, almost all items loaded on the prespecified three factors representing attachment, mentalization and story-self relevance with satisfactory factor loadings and minimal overlaps between item loadings. This indicated that each specified subscale of the ASSP measures a reliable construct, supporting the content validity of the ASSP. The internal consistency of the four pre-defined subscales were shown to be satisfactory (mean *α* = 0.76), providing further evidence for the internal reliability of the ASSP. Therefore, the results yielded by different subscales could in theory reveal meaningful characteristics of specific psychological constructs in adolescents.

The cross-scale correlation between ASSP subscales appeared less straightforward. The overall correlation between ASSP subscales was small (average *r* = 0.23). Besides, unexpected cross-scale correlations, such as the negative correlation between Mentalization and Attachment subscales (*r* = −0.26) were found.

Given that the reliability of the ASSP was supported by the EFA results, the between scale correlations might reveal interesting insights about the complexity of adolescents’ psychological constructs and how better measures could be developed. It is important to note that mentalization, attachment and affect regulation are all complicated constructs, such that the ways the interactive relationships between them are not supposed to be unidimensional (Tanzilli et al., 2021). A good overall mentalization capacity, for example, requires the agent to simultaneously keep context-appropriate balances along four axes, namely automatic (fast, requires little consciousness) versus controlled mentalizing, mentalizing self-versus others, using external (e.g., facial expression) versus internal (e.g., assumptions about the mental state) features, as well as cognitive knowledge versus affective experience for mentalization (Fonagy and Luyten, 2018).

Most items in the ASSP mentalization subscale examined how accurate participants could discriminate the in-the-moment feelings of the animated characters during the conflicts, thereby testing the participants’ ability to recognize the affective mental states of themselves and others. Arguably, participants could get the answers correctly by reasoning from how the stories unfold, using mostly their conscious, cognitive mentalizing ability. It could be possible that the inadequate emotion-based psychological functions were covered by the participants’ cognitive ability in the mentalization subscale, but were later exposed in the Affective Competency subscale, leading to the distortions to the expected positive association between mentalization and emotion regulation capacities. Indeed, in a large study involving over 11,800 adolescents, language ability, which was positively related to other cognitive abilities such as executive function and working memory, were found to be positively correlated internalizing behaviors (Moore and Conway, 2023), indicating that the relationships between various psychological constructs as well as between psychological functions and psychological difficulties could be more complex than what could be seen in unidimensional correlation analyses.

Meanwhile, the results suggested that it would be helpful to decompose different psychological constructs into lower level, fine-grained functional aspects that are minimally dependent on other psychological constructs to ensure the clarity and accuracy when assessing them. The clinical implications behind this are also critical. Many psychological difficulties are highly heterogeneous (i.e., different individuals with the same diagnosis/presentation could have various underlying maladaptive psychological processes; Feczko et al., 2019. Having measures that can provide a detailed insight into young people’s psychological functions would be beneficial for defining a more precise nosology, therefore revealing the best treatment targets that can lead to higher treatment efficacy (Wardenaar and de Jonge, 2013).

Another unexpected finding of the current study was that the convergent validity of the ASSP Mentalization subscale was not confirmed by the correlation between the scores of ASSP subscales and RFQY-5, given the insignificant correlations and very small average *r* = 0.06. This could be interpreted from different perspectives.

Firstly, there exists considerable differences in the nature of the ASSP and the RFQY-5. The RFQY-5 accesses various aspects of participants’ mentalizing profile directly using straightforward, descriptive statements (e.g., I pay attention to my feelings; I’m often curious about the meaning behind others’ actions etc.). On the other hand, the ASSP adopts a more indirect method by asking participants about their thoughts on different imaginative figures in the story. While the brevity and lack of context of the RFQY-5 could make its questions less comprehensive for some participants, it could also be argued that the way ASSP and other story stem measures ask questions itself already requires a certain level of ability to mentalize others, such that it can be difficult to tell which aspects of mentalization were tested by different ASSP questions. Besides, although with a considerable amount of evidence supporting the RFQY-5’s psychometric properties, there were also findings against its reliability (Jewell et al., 2024). Consequently, it would be hard to conclude whether the uncorrelated results were caused by the inadequate psychometric properties of one or both measures, or by the possible fact that the ASSP and RFQY-5 were examining different psychological constructs. The difficulty in finding appropriate measures that could provide firm evidence about the psychometric properties of the ASSP again pointed to the current research gap in effectively examining high level cognitive functions in children and young people. The significance of developing valid and reliable measures that are easy to be administrated in the developing cohort was also highlighted.

### 4.2 Adolescents’ psychological constructs revealed by ASSP

In the Mentalization subscale, low-risk adolescents were found to choose positive mentalization responses more frequently than high-risk adolescents. This resonated with previous research findings that unhealthy mentalizing styles were correlated with maltreatment during early years and could be predictive of higher risks for a range of psychological difficulties (Huang et al., 2020). However, no significant difference was found in the two group’s total scores and their ability in correctly recognizing emotion in themselves. Moreover, the high-risk group outperformed the low-risk group in accurately recognizing emotion in their parents and peers. Several reasons might be at play for these results.

Research indicates that clinically anxious children show heightened neural responses to emotional stimuli of all valences. This reflects their hypervigilant regulatory styles when facing potential threats (Hum et al., 2013). Similarly, Qualter et al. (2013) found that lonely children are hypersensitive to socially threatening stimuli. The high-risk group in this study consists of young individuals in foster care, who often experience early adverse events, making them more vulnerable to anxiety and loneliness. Consequently, they may allocate more neural resources to recognizing emotions, especially negative and threatening cues from others (Masten et al., 2008). Importantly, enhanced accuracy in emotion recognition among parents and peers, compared to typically developing children, can indicate a greater risk for various forms of psychopathology (Suzuki et al., 2015). Therefore, the increased sensitivity of high-risk youth to the emotions of parents and peers—particularly negative emotions—should be a key focus for treatment and early intervention strategies. This approach may help reduce the risk of developing psychopathology.

From another perspective, it was notable that both groups’ scores in themes regarding emotion recognition were very high (Mean_low-risk_ = 1.81, Mean_high-risk_ = 1.90 out of the full score of 2.00), such that there could be limitations in the sensitivity and coverage of these themes at the top-scoring end of the scale (Terwee et al., 2007). The high ceiling effect might represent the inability of these themes in effectively and accurately distinguishing participants with different functional levels in emotion recognition (Bernstein Houck and Hammer, 2019). Future studies could further investigate whether and in what way adolescents with different levels of risks for psychological difficulties have a real functional difference in emotion recognition by (1) increasing the difficulty of questions in ASSP themes a1-a3 and (2) examining whether there are differences in their ability to recognize positive, neutral and negative emotions.

The low-risk group’s significantly higher total score in the Attachment subscale indicated that healthier attachment responses could be found in adolescents with lower risks for psychological difficulty, providing evidence for the validity of the attachment theory and the ASSP in distinguishing young people with various levels of risks for psychological difficulty (Goossens et al., 1998). The low-risk group scored higher in all themes under Attachment subscale, nonetheless, no significant difference was detected. Similar to patterns observed in the Mentalization subscale, the scores of both groups were closely clustered around the midrange of the Attachment scale (between 2 and 2.5 out of the total score of 5). This pattern implies that the Attachment subscale might not effectively differentiate participants with varying risk levels, thus potentially hindering its ability to accurately indicate the participants’ vulnerability to psychological difficulties.

Additionally, it is worth mentioning that differences in the two group’s frequency in choosing secure attachment responses was borderline insignificant (*p* = 0.08). Combining with the significant difference in the two group’s positive mentalization frequency, meaningful insights regarding how questions could be phrased in story stem measures to improve discriminant validity could be shown. Specifically, significant differences in the two risk groups’ mentalization and attachment styles could be demonstrated by their different performances in questions queried on the categorical mentalizing or attachment responses (in which the available options are like She/He could explain why they did what they did; The parents went up to her/his room and Alex told them how she/he was feeling etc.). These types of questions could be more relatable for the participating young people, thus more effectively activating their internal working models. Therefore, phrasing options in story stem measures into in-context, categorical behavioral responses could be more useful for demonstrating young people’s psychological profile, thereby increasing the measure’s validity.

In the Affective Competency subscale, the high-risk group scored significantly higher than the low-risk group for their total score and affect regulation speed. However, higher scores in this subscale do not necessarily indicate better affect regulation. Adaptive emotion regulation skills were proposed to develop within the context of early parent–child relationships, in which attachment quality and co-regulatory functions of caregivers are essential building blocks for the young people’s independent emotion regulatory strategies (Eisenberg et al., 2010). Adolescents in foster care often lacked these positive contexts, thus have more dysregulated affects in various forms. The significantly faster affect regulation speed of the high-risk group could be a result of their adaptation to highly volatile, non-predictable and frightening environments (Rogosch et al., 1995). Alternatively, it could reflect the tendency to supress emotion expression after conflicts that was observed in foster care children (Maughan and Cicchetti, 2002). Higher in-the-moment emotional arousal during conflicts of the high-risk group was also captured by their higher scores for theme c2, potentially suggesting their impaired ability to regulate intense emotional arousal (Villalta et al., 2018), albeit the difference was not statistically significant.

No significant difference between the two risk groups’ responses was found in the Story-self Relevance subscale. This was not surprising as all conflicts depicted in the ASSP are common scenarios that could happen to all young people, including no particularly negative events such as physical or mental abuse and neglect. The differences between the two risk groups’ psychological constructs were supposed to be reflected by their responses and regulatory strategies toward these ordinary problems that they could easily encounter in their daily lives. This corresponds to the theory that the vulnerability of adolescents to mental health problems is closely linked to their various adaptability to the substantial changes that are universally experienced by every young people in physiological and psychological aspects during that developmental stage (Blakemore, 2019). For this specific subscale, it could be seen from their average score (2.00 out of the total score of 5.00) that stories in the ASSP could remind participating adolescents of their own experiences, but not to the extent that they bring the adolescents back to their traumatizing memories in an intolerable way. This could support the superiority of the ASSP, as a story stem measure, that it is comparably less intrusive and could therefore cause less disengagement due to uncomfortable feelings triggered by the questions (Nadar, 2011).

A clear pattern of how risks for psychological difficulty can increase given early childhood adversity, lack of healthy family environment and thus maladaptive psychological constructs could be seen when combining the results of the four ASSP subscales together. Specifically, insecure attachment styles indicated by the lower performance of the high-risk group in the Attachment subscale could be closely associated with their mentalization failures as indicated by their lower positive mentalizing frequency in the Mentalization subscale. In turn, the high-risk adolescents’ inability to adjust their emotions and maintain good psychological functions to overcome the conflicts could be seen in their general lower performance in the Affective Competency, Attachment and Mentalization subscales. This link is proposed and widely supported by the attachment theory that established the rationale basis for the construct of the ASSP. Specifically, individuals with poorer early attachment relationships with more adversity and dysregulated arousals could develop insecure attachment styles and dysfunctional internal working models, which in turn could hinder their reflective functioning and affect regulation (Luyten and Fonagy, 2015), cause failures in solving conflicts using conscious and reflective processes (Lieberman, 2007). Therefore, the results again highlighted the importance of the good quality of early care-giving environment and attachment, and suggested attachment, mentalization, and affective competencies as core targets for assessing, preventing and treating mental health problems in adolescents.

### 4.3 Limitations and future directions

This study examined the psychometric properties of the ASSP, provided useful suggestions for more appropriate psychological measures to be developed for adolescents while deepening the insights into adolescents’ internal constructs. We demonstrated that the ASSP had considerable reliability and validity, and offered insights regarding how the ASSP and other story-stem measures can be improved for future clinical and research purposes. While the psychological processes behind some aspects of the participants’ responses to the ASSP remained ambiguous, this meaningfully demonstrated the complexity of adolescents’ psychological construct which necessitates the use of more comprehensive measures rather than simple self-report questionnaires.

However, several limitations also exist. Although the overall sample size of this study was satisfactory, the size of the two risk groups was disproportionate due to difficulties in collecting high-risk data. Consequently, the sample size of the high-risk sample was not sufficiently large which would have enabled us to examine the impact of different pre-placement risk factors and discontinuities in care. In addition, the data collection of the low-risk sample was conducted in secondary schools by schoolteachers rather than by professional psychologists. Such data collection context may have impacted the depth of information gathered, including detailed demographic information which we were unable to collect more data on. Consequently, there lacked enough evenly distributed data across age, gender and ethnicity backgrounds for demographically matched group comparisons and relevant mediation analyses. This should be emphasized in future studies given the unstable and heterogenetic characteristics of the psychological constructs specifically during adolescence, that it could be helpful to explore how different demographic factors play a role in mediating young people’s thoughts and feelings during this highly volatile period in their development.

Due to limited time and resources, the ASSP’s test–retest reliability and sensitivity to changes across time were not examined in the current study. It would also be desirable to include more previously validated measures, such as the Difficulties in Emotion Regulation Scale (DERS; Hallion et al., 2018), to further explore the construct validity and convergent validity of the ASSP from perspectives in additional to mentalization. Further investigations into the ASSP’s psychometric properties should be done to fully address its strength and weaknesses in assessing adolescents’ internal constructs, thereby improving the quality of the ASSP and providing further suggestions for the development of story stem measures in general.

## 5 Conclusion

An integrative way to understand and assess the psychological construct of adolescents has been long lacking, bringing barriers in effectively preventing, detecting and treating mental health problems in young people. By introducing the attachment theory and relevant concepts that established the development of the ASSP, as well as experimentally examining how adolescents of different levels of risks for psychological difficulty responded to the ASSP questions, this study provided a meaningful insight about the psychometric properties of the ASSP and built the first step toward the validation and application of the ASSP. The results of this study highlighted the current knowledge gap in the complex associations and interactions between different psychological functions in adolescents, as well as how they are linked to psychological difficulty. Meanwhile, it proposed story stem measures as a potentially handy way to effectively explore adolescents’ internal constructs and fill the knowledge gap. Specifically, with the positive evidence for the reliability and validity of the ASSP and the interesting findings regarding adolescent’s psychological functions, the future refinement and practical use of the ASSP appeared promising and valuable.

## Data Availability

The raw data supporting the conclusions of this article will be made available by the authors upon request.
