# Supplementary material for: Preliminary findings on psychometric properties of the adolescent story stem profile
Source: Front Psychol. 2025 Mar 11;16:1478372. doi: 10.3389/fpsyg.2025.1478372 (PMC11933004; doi:10.3389/fpsyg.2025.1478372)
Supplement: Supplementary file 1 [file Data_Sheet_1.docx]

**Appendices for**

**The Adolescent Story Stem Profile: Psychometric Properties and Implications for Adolescents’ Psychological Constructs**

**Zhang, Y., Hillman, S. , Pereira, M. , Anderson, K. , and Cross, R. (2024)**

## **Appendix A**

## **ASSP stories and sample questions.**

Story 1: Alex suddenly walked out of the room and went up to their bedroom.

1a How do you think Alex was feeling when in the room with his parents?

1b How did Alex's parents feel?

1c Why did Alex leave the room?

1d What do you think happened?

1e How do you think Alex was feeling when he was in his

own room?

1f When Alex was in his room, what did his parents do?

1g Did Alex talk to his parents?

1h How much did his parents know that he was upset?

1i What do you think Alex made of the whole situation?

Story 2: Alex is given a certificate at school and goes home with it.

2a How do you think Alex was feeling when given the

certificate?

2b How do you think Alex's friends might have acted?

2c Did Alex talk to his friends about what he had done?

2d How much did Alex's friends know what he was feeling?

2e How did Alex's mother feel?

2f Did Alex talk to his parents about what happened at

school?

2g How much did Alex's parents know how he was feeling?

2h How do you think Alex was feeling about the whole thing?

Story 3: Alex gets suspended from school and then comes home.

3a How do you think Alex was feeling when he was given the

suspension?

3b How do you think Alex's friends might have acted?

3c Did Alex talk to his friends about the suspension?

3d How much did Alex's friends know what he was feeling?

3e When Alex got home, who did he talk to about the

suspension?

3f How did Alex's mother feel upon finding out?

3g How did Alex's father feel on finding out?

3h How much did Alex's parents know what he was feeling?

3i What actions do you think Alex's parents took?

3j How was Alex feeling about being suspended?

3k What do you think Alex made of the whole situation?

Story 4: Alex approaches his friends, asking them like to do something together. While one friend agrees, the other friend says they are busy doing something else.

4a How did Alex feel?

4b How did the friend that wanted Alex to join them feel?

4c How did the friend that didn't want Alex to join them feel?

4d How do you think Alex felt after the conversation?

4e What do you think Alex did?

4f What did Alex do next?

4g Did Alex talk to anyone after this happened?

4h How much did the others know what Alex was feeling?

4i Did Alex show the others how he was feeling?

4j What do you think Alex felt about the situation?

4k What do you think happened in the end in the story?

4l What do you think Alex made of the whole situation?

Story 5: Alex's friends came round and he went out with them. At the end, something has happened to Alex.

5a How do you think Alex was feeling when he went out?

5b How did his parents feel?

5c Where do you think Alex went in the end?

5d What do you think happened to Alex?

5e How do you think Alex was feeling at the end?

5f How do you think Alex's friends were feeling at the end?

5g What did Alex's friends do?

5h Did Alex's friends know what he was feeling?

5i Did Alex talk to his friends?

5j Did Alex's parents know what happened?

5k Did Alex talk to his parents?

5l How did Alex's parents feel when they found out?

5m Why do you think Alex went out to wherever he went?

5n What happened in the end?

5o What do you think Alex made of the whole situation?

5p What do you think Alex was feeling about what happened?

Story 6: Alex has just heard his parents having an argument.

6a How do you think Alex was feeling when in the room with

his parents?

6b How did Alex's Mum feel?

6c How did Alex's Dad feel?

6d What did Alex do?

6e Did his parents know how Alex was feeling?

6f Did Alex talk to his parents about what he felt?

6g Did his Mum talk to Alex about the argument?

6h Did his Dad talk to Alex about the argument?

6i Did Alex talk to his friends about what he felt?

6j What happened about the argument in the end?

6k What do you think Alex was feeling about what happened?

6k How did Alex feel in the end?

6l What did Alex make of the whole thing?

6m What happened with the argument in the end?

Affect Question Response Categories

Happy Funny Excited Proud Supportive Understanding Praising

Not bothered Confused Surprised Uncertain Backed off

Ashamed Guilty Sorry Frightened Worried Sad Upset Troubled

Angry Disgusted Bothered Envious Jealous Aggressive Bullying Teasing Frustrated

Mentalization Question ResponseCategories

Self-mentalizing

He just felt it was his own fault

He wished he had done better - it was not good enough

He just felt it was his own fault

He blamed himself for the whole thing

He just felt angry that it was his own fault

He regretted and blamed himself for all of it

Other-mentalizing

He just felt really annoyed with his parents

He thought others in his class were not so clever so it was no big deal.

He just felt angry with his teachers / the school

He felt angry with his friend Jamie

He just felt really angry with his friends

He couldn't stop feeling bad for his parents arguing

Excessive

He just couldn't t stop thinking about what happened

He couldn't stop feeling happy and telling everyone

He just couldn't stop thinking about what happened

He just couldn't stop thinking about what happened

He just couldn't stop thinking about what happened

He hated his Mum and/or Dad

Reflective

He just wished he had talked to them about the problem

He was clever and worked hard for it

He just wished he had behaved differently

He just wished he had tried harder to get his friend to invite Ruby

He just wished he had been more careful

He was sorry and wanted to help make things better

Under-mentalizing

He felt there was nothing else he could do

He was lucky and it wasn't that difficult

He felt there was nothing else he could do

There was nothing else he could do about it

There was nothing else he could do about it

There was nothing else he could do about it

Attachment Question Response Categories

Dismissing other

He did not want to be with his parents

He didn't like school

He didn't want to go out with them

He didn't want to go out with them

He didn't want to stay in with his parents

He didn't want to stay in with his parents arguing

Preoccupied other

He just couldn't bear to be in the same room as his family

He wanted to get suspended as he has always hated school

He felt the other boys(s) were so horrible he would have to get his own back

He felt the boy Jamie was so horrible he would have to get his own back

He felt so angry with his friends and/or parents for what happened

He felt so angry with his parents for arguing

Preoccupied self

He regretted what he had done and couldn't stop thinking about it

He wanted to get suspended so he could be at home more

He couldn't stop feeling upset that this happened to him

He couldn't stop feeling upset that this happened to him

He couldn't stop feeling upset that this happens to him

He was upset that his parents didn't care about him

Secure/ organised

He needed to calm down as he was upset

He regretted what he had done and worried about his parents' reaction

He could have expressed how he was feeling about it

He could have expressed how he was feeling about it

He was thinking about how others would be feeling about what had happened

He was thinking about how his parents would be feeling about what had happened

Avoidant

He did not have anything to say

He was unlucky that it was him

He could have forgotten about it

He could have forgotten about it

He just forgot about it

He just forgot about it

Disorganised

He was frightened of his parents

He blamed his parents for being suspended

He actually hated both of these boys

He actually hates his friend Ruby

He was frightened of his friends

He loved his parents arguing

## **Appendix B**

## **Full factor loading table for the exploratory factor analysis.**

|  | **Factor** **1**  ‘Story-self relevance’  subscale D  10% | **Factor** **2**  ‘Attachment’  subscale B  18% | **Factor** **3**  ‘mentalization’  subscale A and C  24% |
| --- | --- | --- | --- |
| **s1a11** |  |  |  |
| **s1a3** |  |  |  |
| **s1a12** |  |  |  |
| **s1b21** |  |  |  |
| **s1b22** |  | 0.319 |  |
| **s1b11** |  |  |  |
| **s1b12** |  | 0.307 |  |
| **s1c1** |  |  |  |
| **s1d1** | 0.59 |  |  |
| **s1d2** | 0.545 |  |  |
| **s2a11** |  |  | 0.334 |
| **s2c2** |  |  |  |
| **s2a13** |  |  | 0.385 |
| **s2b11** |  | 0.555 |  |
| **s2b12** |  | 0.523 |  |
| **s2a12** |  |  | 0.478 |
| **s2b21** |  | 0.585 |  |
| **s2b22** |  | 0.641 |  |
| **s2c1** |  |  |  |
| **s2d1** | 0.71 |  |  |
| **s2d2** | 0.62 |  |  |
| **s3a11** |  |  |  |
| **s3c2** |  |  |  |
| **s3a13** |  |  | 0.359 |
| **s3b11** |  | 0.538 |  |
| **s3b12** |  | 0.484 |  |
| **s3a12** |  |  | 0.62 |
| **s3b21** |  | 0.514 |  |
| **s3b22** |  | 0.504 |  |
| **s3c1** |  |  | -0.42 |
| **s3d1** | 0.721 |  |  |
| **s3d2** | 0.637 |  |  |
| **s4a11** |  |  |  |
| **s4c2** |  |  | 0.335 |
| **s4a13** |  |  |  |
| **s4b11** |  | 0.419 |  |
| **s4b12** |  | 0.378 |  |
| **s4b21** |  | 0.337 |  |
| **s4b22** |  | 0.441 |  |
| **s4c1** |  |  | -0.403 |
| **s4d1** | 0.752 |  |  |
| **s4d2** | 0.631 |  |  |
| **s5a11** |  |  | 0.608 |
| **s5c2** |  |  | 0.411 |
| **s5a13** |  |  | 0.546 |
| **s5b11** |  | 0.345 |  |
| **s5b12** |  | 0.345 |  |
| **s5a12** |  |  | 0.49 |
| **s5b21** |  | 0.361 |  |
| **s5b22** |  | 0.452 |  |
| **s5c1** |  |  |  |
| **s5d1** | 0.778 |  |  |
| **s5d2** | 0.727 |  |  |
| **s6a11** |  |  | 0.6 |
| **s6c2** |  |  | 0.369 |
| **s6a12** |  |  | 0.496 |
| **s6b21** |  | 0.369 |  |
| **s6b22** |  | 0.364 |  |
| **s6b11** |  |  |  |
| **s6b12** |  |  |  |
| **s6c1** |  |  | -0.308 |
| **s6d1** | 0.706 |  |  |
| **s6d2** | 0.656 |  |  |
